# Messung der körperlichen Fitness in der NAKO Gesundheitsstudie – Methoden, Qualitätssicherung und erste deskriptive Ergebnisse

**DOI:** 10.1007/s00103-020-03100-3

**Published:** 2020-02-18

**Authors:** Alexander Kluttig, Johannes Zschocke, Johannes Haerting, Axel Schmermund, Sylvia Gastell, Karen Steindorf, Florian Herbolsheimer, Andrea Hillreiner, Carmen Jochem, Sebastian Baumeister, Ole Sprengeler, Tobias Pischon, Lina Jaeschke, Karin B. Michels, Lilian Krist, Halina Greiser, Gerhard Schmidt, Wolfgang Lieb, Sabina Waniek, Heiko Becher, Annika Jagodzinski, Sabine Schipf, Henry Völzke, Wolfgang Ahrens, Kathrin Günther, Stefanie Castell, Yvonne Kemmling, Nicole Legath, Klaus Berger, Thomas Keil, Julia Fricke, Matthias B. Schulze, Markus Loeffler, Kerstin Wirkner, Oliver Kuß, Tamara Schikowski, Sonja Kalinowski, Andreas Stang, Rudolf Kaaks, Antje Damms Machado, Michael Hoffmeister, Barbara Weber, Claus-Werner Franzke, Sigrid Thierry, Anette Peters, Nadja Kartschmit, Rafael Mikolajczyk, Beate Fischer, Michael Leitzmann, Mirko Brandes

**Affiliations:** 1 Institut für Medizinische Epidemiologie, Biometrie und Informatik, Martin-Luther-Universität Halle-Wittenberg, Magdeburger Str. 8, 06112 Halle (Saale), Deutschland; 2 Institut für Physik, Martin-Luther-Universität Halle-Wittenberg, Halle (Saale), Deutschland; 3 Bethanien Hospital, Frankfurt, Deutschland; 4 NAKO Studienzentrum, Deutsches Institut für Ernährungsforschung, Potsdam-Rehbrücke, Deutschland; 5 Abteilung Bewegung, Präventionsforschung und Krebs, Deutsches Krebsforschungszentrum (DKFZ), Heidelberg, Deutschland; 6 Institut für Epidemiologie und Präventivmedizin, Universität Regensburg, Regensburg, Deutschland; 7 Lehrstuhl für Epidemiologie der LMU München, UNIKA-T, Augsburg, Deutschland; 8 BIPS, Leibniz Institut für Präventionsforschung und Epidemiologie, Bremen, Deutschland; 9 Forschergruppe Molekulare Epidemiologie, Max-Delbrück-Centrum für Molekulare Medizin in der Helmholtz-Gemeinschaft (MDC), Berlin, Deutschland; 10 Institut für Prävention und Tumorepidemiologie, Universitätsklinikum Freiburg, Medizinische Fakultät, Albert-Ludwigs-Universität Freiburg, Freiburg, Deutschland; 11 Institut für Sozialmedizin, Epidemiologie und Gesundheitsökonomie, Charité – Universitätsmedizin Berlin, Berlin, Deutschland; 12 Hochschule Kaiserslautern, Zweibrücken, Deutschland; 13 Institut für Epidemiologie, Christian-Albrechts-Universität Kiel, Kiel, Deutschland; 14 Institut für Medizinische Biometrie und Epidemiologie, Universitätsklinikum Hamburg-Eppendorf, Hamburg, Deutschland; 15 Epidemiologisches Studienzentrum, Universitätsklinikum Hamburg-Eppendorf, Hamburg, Deutschland; 16 Institut für Community Medicine, Universitätsmedizin Greifswald, Greifswald, Deutschland; 17 Helmholtz-Zentrum für Infektionsforschung (HZI), Braunschweig, Deutschland; 18 Institut für Epidemiologie und Sozialmedizin, Universität Münster, Münster, Deutschland; 19 Institut für Medizinische Informatik, Statistik und Epidemiologie (IMISE), Universität Leipzig, Leipzig, Deutschland; 20 Institut für Biometrie und Epidemiologie, Deutsches Diabetes-Zentrum (DDZ), Leibniz-Zentrum für Diabetes-Forschung an der Heinrich-Heine-Universität Düsseldorf, Düsseldorf, Deutschland; 21 IUF – Leibniz-Institut für umweltmedizinische Forschung, Düsseldorf, Deutschland; 22 Institut für Medizinische Informatik, Biometrie und Epidemiologie (IMIBE), Universitätsklinikum Essen, Essen, Deutschland; 23 Institut für Epidemiologie, Helmholtz Zentrum München, Neuherberg, Deutschland; 24 LIFE – Leipziger Forschungszentrum für Zivilisationserkrankungen, Universität Leipzig, Leipzig, Deutschland; 25 Institut für Statistik, Fachbereich Mathematik und Informatik, Universität Bremen, Bremen, Deutschland; 26 Charité – Universitätsmedizin Berlin, Berlin, Deutschland; 27 MDC/BIH Biobank, Max-Delbrück-Centrum für Molekulare Medizin in der Helmholtz-Gemeinschaft (MDC) und Berlin Institute of Health (BIH), Berlin, Deutschland; 28 Partnerstandort Berlin, Deutsches Zentrum für Herz-Kreislauf-Forschung (DZHK), Berlin, Deutschland; 29 Abteilung Molekulare Epidemiologie, Deutsches Institut für Ernährungsforschung, (DIfE), Nuthetal, Deutschland; 30 Institut für Klinische Epidemiologie und Biometrie, Universität Würzburg, Würzburg, Deutschland; 31 Landesinstitut für Gesundheit, Bayerisches Landesamt für Gesundheit und Lebensmittelsicherheit, Bad Kissingen, Deutschland; 32 Krebsregister Saarland, Saarbrücken, Deutschland; 33 Deutsches Krebsforschungszentrum (DKFZ), Heidelberg, Deutschland; 34 Selbstständige Forschungsgruppe Klinische Epidemiologie, Helmholtz Zentrum München, Deutsches Forschungszentrum für Gesundheit und Umwelt, München, Deutschland; 35 Abteilung Klinische Epidemiologie und Alternsforschung, Deutsches Krebsforschungszentrum (DKFZ), Heidelberg, Deutschland; 36 Partnerstandort Greifswald, Deutsches Zentrum für Herz-Kreislauf-Forschung (DZHK), Greifswald, Deutschland; 37 Partnerstandort Hamburg, Deutsches Zentrum für Herz-Kreislauf-Forschung (DZHK), Hamburg, Deutschland

**Keywords:** Körperliche Leistungsfähigkeit, Kraft, Greifkraft, Kardiorespiratorische Fitness, NAKO Gesundheitsstudie, Physical fitness, Muscle strength, Grip strength, Cardiorespiratory fitness, German National Cohort

## Abstract

Die körperliche Fitness ist das Maß für die individuelle Fähigkeit, körperlich aktiv zu sein. Ihre wesentlichen Komponenten sind die kardiorespiratorische Fitness (Cardiorespiratory Fitness, CRF), die Muskelkraft und die Beweglichkeit. Neben der körperlichen Aktivität ist die körperliche Fitness ein wesentlicher Prädiktor für Morbidität und Mortalität.

Ziel der Arbeit sind die Beschreibung der Erhebungsmethoden körperlicher Fitness in der NAKO Gesundheitsstudie und die Darstellung erster deskriptiver Ergebnisse.

In der NAKO-Basiserhebung wurden die maximale Handgreifkraft (Grip Strength, GS) und die CRF als Komponenten der körperlichen Fitness über ein Handdynamometer bzw. über einen Fahrradergometertest mit submaximaler Belastung erhoben. Daraus wurde die maximale Sauerstoffaufnahme (VO_2max_) zur Beurteilung der CRF abgeleitet. Die Ergebnisse von insgesamt 99.068 GS-Messungen und 3094 Messungen der CRF beruhen auf einem Datensatz zur Halbzeit der Basiserhebung der NAKO (Alter 20–73 Jahre, 47 % Männer).

Männer zeigten im Vergleich zu Frauen höhere Werte der körperlichen Fitness (Männer: GS = 47,8 kg, VO_2max_ = 36,4 ml·min^−1^ · kg^−1^; Frauen: GS = 29,9 kg, VO_2max_ = 32,3 ml·min^−1^ · kg^−1^). Ungefähr ab dem 50. Lebensjahr konnte ein Rückgang der GS verzeichnet werden, wohingegen die CRF ab der Altersgruppe 20–29 Jahre bis zu den ≥60-Jährigen kontinuierlich abfiel. Die GS und die VO_2max_ zeigten nach Korrektur für das Körpergewicht einen linear positiven Zusammenhang (Männer β = 0,21; Frauen β = 0,35).

Die Analysen zeigten eine gute Übereinstimmung der Verteilung der körperlichen Fitness in der NAKO im Vergleich zu anderen bevölkerungsbasierten Studien. Zukünftige Auswertungen werden insbesondere die unabhängige Bedeutung der GS und CRF bei der Prädiktion von Morbidität und Mortalität beleuchten.

## Abstract (en)

Physical fitness is defined as an individual’s ability to be physically active. The main components are cardiorespiratory fitness (CRF), muscle strength, and flexibility. Regardless of physical activity level, physical fitness is an important determinant of morbidity and mortality.

The aim of the current study was to describe the physical fitness assessment methodology in the German National Cohort (NAKO) and to present initial descriptive results in a subsample of the cohort.

In the NAKO, hand grip strength (GS) and CRF as physical fitness components were assessed at baseline using a hand dynamometer and a submaximal bicycle ergometer test, respectively. Maximum oxygen uptake (VO_2max_) was estimated as a result of the bicycle ergometer test. The results of a total of 99,068 GS measurements and 3094 CRF measurements are based on a data set at halftime of the NAKO baseline survey (age 20–73 years, 47% men).

Males showed higher values of physical fitness compared to women (males: GS = 47.8 kg, VO_2max_ = 36.4 ml·min^−1^ · kg^−1^; females: GS = 29.9 kg, VO_2max_ = 32.3 ml · min^−1^ · kg^−1^). GS declined from the age of 50 onwards, whereas VO_2max_ levels decreased continuously between the age groups of 20–29 and ≥60 years. GS and VO_2max_ showed a linear positive association after adjustment for body weight (males β = 0.21; females β = 0.35).

These results indicate that the physical fitness measured in the NAKO are comparable to other population-based studies. Future analyses in this study will focus on examining the independent relations of GS and CRF with risk of morbidity and mortality.

## Einleitung

Der körperlichen Fitness wird eine besondere Rolle für die Gesundheit des Menschen zugesprochen. Dabei umfasst die gesundheitsbezogene körperliche Fitness im Allgemeinen die Leistungsfähigkeit des Herz-Kreislauf-Systems (Cardiorespiratory Fitness, CRF), die Muskelausdauer, die Muskelkraft, die Körperzusammensetzung sowie die Beweglichkeit [1]. Von diesen Komponenten der körperlichen Fitness haben sich insbesondere die Muskelkraft sowie die CRF als bedeutungsvolle prognostische Indikatoren für die Gesundheit des Menschen erwiesen. Bezogen auf die Muskelkraft konnte gezeigt werden, dass die Handgreifkraft (Grip Strength, GS) ein sehr guter Parameter für die Vorhersage von Gesundheitsrisiken ist [2–4]. So konnte z. B. in der PURE-Studie ein inverser Zusammenhang zwischen der GS und der allgemeinen und kardiovaskulären Mortalität gezeigt werden [2]. Eine reduzierte GS ist insbesondere bei älteren Erwachsenen mit einer Reduktion der Lebensqualität und funktionellen Einschränkungen im Alltag assoziiert [5–7].

Lee et al. zeigen, dass sich das allgemeine Mortalitätsrisiko bei Frauen und Männern mit gut ausgeprägter CRF gegenüber Personen mit schlecht ausgeprägter CRF nahezu halbiert. Bezogen auf die kardiovaskuläre Mortalität wurde bei Frauen mit gut ausgeprägter CRF nur noch ein Drittel des Risikos, vorzeitig an einer Herz-Kreislauf-Erkrankung zu sterben, festgestellt [8]. Begründet ist die hohe Vorhersagekraft der CRF durch ihren nachgewiesenen Einfluss auf Herz-Kreislauf-Erkrankungen [9, 10], Krebs [11] und Diabetes [12] sowie auf die mentale Gesundheit [13]. Als Goldstandard zur Bestimmung der CRF wird die maximale Sauerstoffaufnahme (VO_2max_) angesehen, die idealerweise über eine Spiroergometrie bis zur subjektiven Ausbelastung gemessen wird [14]. Ist eine maximale Belastung z. B. aus gesundheitlichen oder organisatorischen Gründen nicht möglich, kann die VO_2max_ anhand der Herzfrequenz (HF) oder des subjektiven Belastungsempfindens in einem submaximalen Test bestimmt werden [15, 16].

Die NAKO Gesundheitsstudie ist mit über 200.000 Teilnehmenden die derzeit größte bevölkerungsbezogene Kohortenstudie in Deutschland [17]. Im Rahmen der Zielsetzung der NAKO stellt die Erhebung der körperlichen Fitness einen zentralen Baustein dar. Der vorliegende Beitrag beschreibt, wie die körperliche Fitness über die Messung von CRF und GS in das Studiendesign der NAKO implementiert wurde. Des Weiteren werden die Maßnahmen zur Qualitätssicherung der erhobenen Daten sowie erste deskriptive Ergebnisse zur körperlichen Fitness der NAKO-Studienteilnehmenden dargestellt.

## Methoden

### Studiendesign

Details zur NAKO und zur Datenerhebung können in der Publikation zum Design der Studie nachgelesen werden [17]. Insgesamt mehr als 200.000 Personen im Alter von 20–69 Jahren (Alter zum Zeitpunkt der Stichprobenziehung) nahmen an der Basiserhebung der Studie teil. Bei allen Teilnehmenden wurde ein ca. 3,5-Stunden dauerndes Untersuchungsprogramm durchgeführt (Level 1). Zufällig ausgewählte 20 % der Teilnehmenden erhielten ein ca. 6 h dauerndes, ausführlicheres Untersuchungsprogramm (Level 2). Die vorliegende Analyse beruht auf einem Datensatz zur Halbzeit der Basiserhebung der NAKO (siehe Beitrag von Schipf et al. in diesem Themenheft). Eingeschlossen wurden alle Teilnehmenden, die vom 14.03.2014 bis zum 17.03.2017 im Rahmen der NAKO Basiserhebung untersucht wurden (*N* = 101.734).

Alle Teilnehmenden wurden zu Beginn der Untersuchung ausführlich über die Studienziele, das Untersuchungsprogramm und datenschutzrelevante Belange im Rahmen des modularen Einwilligungsprozesses informiert, eine informierte schriftliche Einwilligungserklärung wurde eingeholt. Das Studienprotokoll der NAKO wurde von den Ethikkommissionen der teilnehmenden Einrichtungen sowie vom Bundesbeauftragten für Datenschutz und Informationsfreiheit geprüft und positiv beurteilt.

### Erfassung der körperlichen Fitness

#### Handgreifkraft (GS)

Die Messung der maximalen isometrischen GS erfolgte im Rahmen der Level-1-Untersuchung bei allen Teilnehmenden mit dem Jamar Plus+ Greifkraftdynamometern (Sammons Preston, Rolyon, Bolingbrook, IL, USA). Die Geräte wurden alle zwei Jahre durch den Hersteller kalibriert und im Abstand von 6 Wochen in den Studienzentren auf Messgenauigkeit mit Eichgewichten überprüft. Regelmäßig fanden Überprüfungen der Untersucher- und Geräteeinflüsse auf die Untersuchungsergebnisse der GS-Messung statt. Auffällige Untersuchende wurden entsprechend nachgeschult, auffällige Geräte hinsichtlich ihrer Messgenauigkeit überprüft und ggf. rekalibriert oder ausgetauscht.

Die Messung der GS erfolgte in der Standardposition für Greifkraftmessungen [18] in aufrecht sitzender Position auf einem Stuhl ohne Armlehnen, mit den Füßen auf dem Boden, neutraler Schulterposition, ca. 90° Armbeuge und neutraler Unterarmposition mit je drei Versuchen abwechselnd mit beiden Händen. Für alle Teilnehmenden wurde unabhängig von der Handgröße die Griffweite 2 gewählt. Dieses Vorgehen wurde vor Beginn der Studie intern getestet, mit dem Ergebnis, dass die Messung mit einer einzigen Griffweite für alle Teilnehmenden ausreichend valide ist, um die maximale GS zu erfassen [19]. Den Teilnehmenden wurde vor dem Test das Vorgehen ausführlich erklärt, insbesondere dass es das Ziel der Messung ist, die maximal mögliche GS zu erfassen. Die Erfassung der Händigkeit erfolgte über die einfache Frage, mit welcher Hand die Teilnehmenden Brot schneiden bzw. mit einer Schere arbeiten (Antwortmöglichkeiten: rechts, links, beidseitig). Während des Tests wurde auf eine motivierende Anleitung verzichtet, da dies bei der Vielzahl der Untersuchenden der NAKO schwer zu standardisieren gewesen wäre [20]. Ausgeschlossen von der GS-Messung wurden lediglich Personen, bei denen beidseitig akute Verletzungen oder Operationen vorlagen bzw. eine beidseitige Amputation oder Lähmung der Arme. Äußerte ein Teilnehmender Bedenken gegenüber der Messung (Angst vor Schmerzentstehung etc.), wurde ein Testversuch angeboten.

Die Ergebnisse der GS-Messungen wurden direkt von den Untersuchenden in das NAKO-interne, webbasierte Dokumentationssystem eingegeben. Bei der Datenbereinigung wurden GS-Werte ≤0 kg oder ≥90 kg als unplausibel definiert. In der Regel handelte es sich bei diesen Einträgen um Tippfehler oder irrtümlich verwendete Codes für fehlende Werte. Weiterhin wurden Untersuchungen ausgeschlossen, bei denen an beiden Händen jeweils weniger als zwei Messversuche durchgeführt wurden. Für die Auswertung wurde unabhängig von der Handseite der höchste gemessene Wert der GS verwendet [18]. Zusätzlich wurde die relative GS berechnet, indem der höchste gemessene GS-Wert durch das Körpergewicht dividiert wurde. Insgesamt liegen für 99.068 Teilnehmende (97,4 %) auswertbare Messergebnisse für die maximale GS vor. Von diesen wurde bei 97.997 Teilnehmenden die Messung der GS an beiden Händen, bei 1071 Teilnehmenden nur an einer Hand durchgeführt. Bei nicht durchgeführten GS-Messungen waren im Wesentlichen gesundheitliche Gründe (60 %), technisch-organisatorische Gründe (26 %) oder die Verweigerung der Untersuchung durch den Teilnehmenden (12 %) ursächlich.

#### Kardiorespiratorische Fitness (CRF)

Die Fahrradergometrie wurde bei Teilnehmenden mit intensiviertem Untersuchungsprogramm (Level 2) in insgesamt 6 von 18 Studienzentren der NAKO-Basisuntersuchung durch notfallmedizinisch geschultes Studienpersonal (keine direkte ärztliche Überwachung) durchgeführt. Darüber hinaus erfolgte auch in den anderen Studienzentren bei jeweils ca. 100 Teilnehmenden eine Fahrradergometrieuntersuchung.

Als Protokoll des submaximalen Fahrradergometertests zur Erhebung der CRF diente ein adaptiertes WHO-Stufenschema [21]. Ziel war es, dass die Teilnehmenden innerhalb von höchstens vier Belastungsstufen ihre submaximale HF (85 % der maximalen HF) erreichen. Die maximale HF wurde anhand der Formel *HF*_*max*_ *=* *208* *−* *0,7 Alter* nach Tanaka berechnet [22]. Bei aktueller Einnahme von HF-senkenden Medikamenten (Betablockern) wurde die Ziel-HF um 20 Schläge/min reduziert. Die Dauer pro Belastungsstufe betrug 2 min, die Steigerung jeweils 25 W. Die Einstiegslast wurde in Abhängigkeit des Alters, des Geschlechts, des Gewichts und des selbst eingeschätzten Fitnesszustands [23] auf 25 W, 50 W, 75 W oder 100 W festgelegt. Während der Belastungsphase wurden die Teilnehmenden angehalten, die Drehzahl zwischen 60–80 U/min zu halten. Sobald die Teilnehmenden ihre submaximale HF-Grenze für 30 s überschritten hatten, wurde eine abschließende 1‑minütige Erholungsphase eingeleitet, in der die Leistung auf 25 W abgesenkt wurde. Wurde die submaximale HF-Grenze während der vier Belastungsstufen nicht erreicht, wurde nach Beendigung der 4. Stufe ebenfalls die Erholungsphase gestartet. Bei subjektiven Beschwerden der Teilnehmenden oder Erschöpfung wurde der Test vorzeitig beendet.

Die Tests wurden auf einem kalibrierten Fahrradergometer mit integriertem Herzfrequenzempfänger (Ergosana Sana Bike 350F) durchgeführt. Die Herzfrequenz wurde mittels eines Polar-Brustgurts (T31, Polar Electro Oy, Finnland) an der Brustwand der Teilnehmenden abgeleitet. Die Steuersoftware des Ergometers (Dr. Schmidt GmbH, Neunkirchen) wurde eigens für die NAKO entwickelt und erlaubt die kontinuierliche Aufzeichnung von HF, Leistung und Drehzahl mit einer Samplingrate von 1 Hz sowie des subjektiven Anstrengungsempfindens (Received Perception of Exertion, RPE) über die Borg- bzw. RPE-Skala [24].

Vor Durchführung der Fahrradergometrie wurden die Ausschlusskriterien für die Untersuchung abgeklärt. Diese Abklärung fand anhand der Untersuchungsergebnisse aus dem EKG, der Blutdruckmessung, der anthropometrischen Untersuchung und der Befragung mittels des modifizierten Physical Activity Readiness Questionnaire (PAR-Q-Fragebogens; [25, 26]) statt. Direkt von der Fahrradergometrie ausgeschlossen wurden Teilnehmende, bei denen eines der folgenden Charakteristika vorlag: Gewicht >160 kg, Blutdruck ≥180 mm/Hg systolisch und/oder ≥110 mm Hg diastolisch, Ruhe-HF ≥100/min oder selbst berichtete Kontraindikation im PAR-Q-Fragebogen.

Die Entscheidung über den Ausschluss von Teilnehmenden von der Fahrradergometrie oblag dem Studienarzt, sofern ein auffälliger automatischer EKG-Befund (basierend auf dem Modular ECG Analysis System, MEANS [27]) vorlag oder abklärungsbedürftige Befunde aus dem PAR-Q-Fragebogen hervorgingen.

Während der Fahrradergometrie wurden HF, Drehzahl und Leistung kontinuierlich mit einer Samplingrate von 1 Hz aufgezeichnet. Darüber hinaus wurde am Ende jeder Belastungsstufe das subjektive Belastungsempfinden (RPE-Wert) dokumentiert. Die von der Ergometersoftware aufgezeichneten Daten wurden in Textdateien gespeichert und mittels Python 3.7 und SAS 9.4 für die Qualitätssicherung und wissenschaftliche Auswertung bearbeitet. Für die Berechnung der HF-bezogenen Leistungsparameter der CRF mussten die HF-Verläufe von Artefakten bereinigt werden. Zunächst wurden aus dem rohen Herzfrequenzsignal zwei zusätzliche Signale über den Median für 9‑ bzw. 19-Sekunden-Zeitfenster berechnet. Beide Signale sorgen für eine Glättung der HF und somit zur Eliminierung von Frequenzsprüngen. Die automatische Qualitätseinschätzung des HF-Verlaufs erfolgte je Belastungsphase anhand der trendbereinigten Standardabweichung des Rohsignals und der geglätteten Signale. Auffällige HF-Aufzeichnungen wurden anschließend einzeln visuell kontrolliert und ggf. korrigiert oder verworfen. Zur Bestimmung der HF-bezogenen Leistungsparameter wurde der Mittelwert der letzten 30 s jedes Belastungsintervalls verwendet, sofern die Belastungsstufe mindestens 1:30 min dauerte und nicht vorab abgebrochen wurde. Bei weniger als 2 vollständigen Belastungsstufen (Test innerhalb der ersten oder zweiten Belastungsstufe abgebrochen) wurden keine HF-bezogenen Leistungsparameter berechnet. Bei insgesamt 61,7 % der Teilnehmenden mit auswertbarer Fahrradergometriemessung wurden die HF-bezogenen Leistungsparameter aus 4 Belastungsstufen berechnet, bei 28,3 % aus 3 Belastungsstufen und bei 10 % aus 2 Belastungsstufen.

Die Berechnung der HF-bezogenen Leistungsparameter erfolgte zum einen nach dem Prinzip der Physical Work Capacity (PWC; [28]), zum anderen über die geschätzte maximale Sauerstoffaufnahme (VO2_max_). Mittels des PWC-Prinzips wird die Leistung bestimmt, die beim Überschreiten einer definierten HF-Schwelle geleistet wird. Hierbei eignen sich insbesondere relative HF-Schwellen, da damit der altersbedingte Rückgang der HF berücksichtigt werden kann. Die hier berichtete PWC 75 % gibt somit an, welche Leistung (in W) ein Teilnehmender erbringt, wenn er oder sie 75 % der eigenen maximalen Herzfrequenz erreicht.

Die Berechnung der VO2_max_ erfolgte zweistufig: Zunächst wurde die maximale Leistung (in W) anhand der erbrachten Leistung bei submaximaler HF und der altersbasierten maximalen Herzfrequenzgrenze nach Tanaka et al. [28] ermittelt. Mit der geschätzten maximalen Leistung wurde über eine modifizierte und im Rahmen eines Prätests validierten Formel des American College of Sports Medicine [29] die VO2_max_ berechnet: VO2_max_  = 33,5 ml·min^−1^ · kg^−1^ + 12,24 (maximale Leistung in W) (Körpergewicht^−1^ in kg).

Insgesamt wurde bei 3424 Teilnehmenden (3,4 %) eine Fahrradergometrie durchgeführt. Dabei konnte bei 44 Untersuchungen die HF-Verlaufsdatei aus technischen Gründen nicht gesichert werden. Nach automatischer und manueller Qualitätskontrolle wurden 91 Untersuchungen aufgrund qualitativ unzureichender HF-Aufzeichnung oder aufgrund von weniger als 2 vollständigen Belastungsstufen ausgeschlossen. Demnach standen für die vorliegende Auswertung CRF-Angaben von 3289 Teilnehmenden zur Verfügung. Da ausschließlich HF-bezogene Leistungsparameter der CRF in dieser Arbeit verwendet wurden, wurden nachträglich zusätzlich 195 Teilnehmende aufgrund der aktuellen Einnahme von Betablockern ausgeschlossen. Somit standen die Daten der Fahrradergometrie von insgesamt 3094 Teilnehmenden für die Analysen zur Verfügung.

Sofern Teilnehmende aufgrund einer Kontraindikation von der Fahrradergometrie ausgeschlossen werden mussten, war hier im Wesentlichen ein auffälliges EKG allein (15 %), ein auffälliger PAR-Q-Fragebogen (58 %) oder eine Kombination aus beiden (14 %) ursächlich.

### Statistische Auswertung

In der deskriptiven Darstellung wurden für kategoriale Variablen absolute und relative Häufigkeiten berechnet, für kontinuierliche Variablen wurden Mittelwert und Standardabweichung bestimmt. Die Darstellung der Altersverteilung erfolgte anhand von 10-Jahres-Altersgruppen. Zum Zeitpunkt der Stichprobenziehung waren alle Teilnehmenden zwischen 20 und 69 Jahre alt. Aufgrund der zeitlichen Verzögerung zwischen Stichprobenziehung und tatsächlicher Untersuchung waren insgesamt 2971 Teilnehmende (2,9 %) zum Zeitpunkt der Untersuchung ≥70 Jahre (davon 91 % zwischen 70 und 71 Jahre). Diese Teilnehmenden wurden in der Darstellung der körperlichen Fitness nach 10-Jahres-Altersgruppen der Gruppe ≥60 Jahre zugeordnet. Der Zusammenhang von Alter und körperlicher Fitness wurde zusätzlich grafisch durch Scatterplots mit geschlechtsspezifischen Regressionsfunktionen dargestellt, wobei aufgrund des nichtlinearen Zusammenhangs von Alter und GS eine lokal gewichtete Regressionsfunktion (LOESS, Locally Weighted Regression) genutzt wurde. Der Zusammenhang von maximaler GS und CRF wurde mit Adjustierung für das Körpergewicht mittels linearer Regression analysiert. Darüber hinaus wurden Pearson-Korrelationskoeffizienten für den Zusammenhang von maximaler GS und CRF berechnet.

Alle Analysen wurden mit SAS (Version 9.4, The SAS Institute, Cary, NC) durchgeführt.

## Ergebnisse

### Stichprobencharakteristik

Tab. 1 zeigt die Stichprobencharakteristik in Abhängigkeit von den verfügbaren Daten aus GS-Messung und Fahrradergometrie. Der hohe Anteil fehlender Fahrradergometrieuntersuchungen lässt sich dadurch erklären, dass a) die Untersuchung nur bei 20 % (Level 2) der Teilnehmenden in 6 von 18 Studienzentren geplant war und b) die Untersuchung aufgrund von Kontraindikationen, Verweigerung oder fehlenden technischen und organisatorischen Voraussetzungen nicht bei jedem potenziell Teilnehmenden durchgeführt werden konnte. Die Altersverteilung bei Teilnehmenden mit durchgeführter Fahrradergometrie weist auf einen Selektionseffekt hin, da insbesondere ältere Teilnehmende aufgrund von Kontraindikationen von der Fahrradergometrie ausgeschlossen werden mussten.

**Tab. 1:** Stichprobencharakteristik und Anzahl auswertbarer Messungen. Daten der NAKO Gesundheitsstudie zur Halbzeit der Basiserhebung

	Beobachtungen im Datensatz		Beobachtungen mit auswertbarer GS-Messung		Beobachtungen mit auswertbarer Fahrradergometrie	
	*N* = 101.734		*N* = 99.068		*N* = 3094	
*Altersgruppen*	*N* (%)		*N* (%)		*N* (%)	
20–29	7081	(6,96)	6984	(7,05)	306	(9,89)
30–39	9596	(9,43)	9414	(9,50)	382	(12,35)
40–49	22.047	(21,67)	21.581	(21,78)	819	(26,47)
50–59	29.390	(28,89)	28.601	(28,87)	899	(29,06)
≥60	33.620	(33,05)	32.488	(32,79)	688	(22,24)
*Geschlecht*						
Männlich	47.235	(46,43)	46.267	(46,70)	1463	(47,29)
Weiblich	54.499	(53,57)	52.801	(53,30)	1631	(52,71)

*N* Anzahl Beobachtungen, *GS* Handgreifkraft

### Maximale Handgreifkraft

**Abb. 1:**
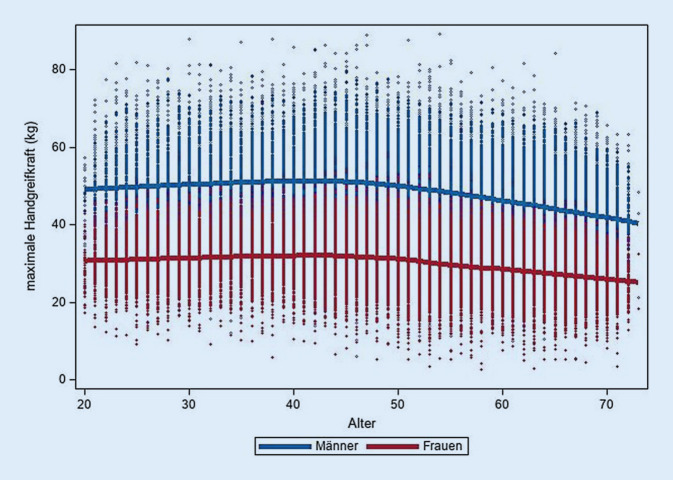
Zusammenhang zwischen Alter und maximaler Handgreifkraft (in kg). Scatterplot mit geschlechtsspezifisch lokal gewichteter Regressionsfunktion (LOESS). Daten der NAKO Gesundheitsstudie zur Halbzeit der Basiserhebung

Die maximale GS betrug bei Männern im Mittel 47,8 kg und bei Frauen 29,9 kg (Tab. 2). Bei beiden Geschlechtern ist ein ähnlicher Alterseinfluss auf die maximale GS zu beobachten mit einem Maximum zwischen dem 30. und 50. Lebensjahr (Tab. 2; Abb. 1). Unter Berücksichtigung des Körpergewichts ist der leichte Anstieg der maximalen GS zwischen dem 20. und 30. Lebensjahr nicht mehr zu beobachten (Tab. 2).

**Tab. 2:** Maximale Handgreifkraft nach Alter und Geschlecht. Daten der NAKO Gesundheitsstudie zur Halbzeit der Basiserhebung

	Männer			Frauen		
Altersgruppen	*N*	Max. GS in kg (SD)	Max. GS relativ in kg/kg Körpergewicht (SD)	*N*	Max. GS in kg (SD)	Max. GS relativ in kg/kg Körpergewicht (SD)
20–29	3008	49,30 (9,57)	0,62 (0,13)	3976	30,98 (5,63)	0,48 (0,11)
30–39	4285	51,56 (9,33)	0,62 (0,12)	5129	32,07 (5,78)	0,48 (0,11)
40–49	9672	51,18 (9,00)	0,60 (0,12)	11.909	32,06 (5,68)	0,47 (0,11)
50–59	13.114	48,46 (8,67)	0,56 (0,12)	15.487	29,86 (5,58)	0,43 (0,11)
≥60	16.188	43,99 (7,99)	0,51 (0,11)	16.300	27,28 (4,98)	0,39 (0,09)
*Gesamt*	46.267	47,81 (9,15)	0,56 (0,12)	52.801	29,86 (5,78)	0,43 (0,11)

*GS* Handgreifkraft, *SD* Standardabweichung, *N* Anzahl Beobachtungen

Bei den teilnehmenden Männern waren 91 % Rechtshänder, 6 % Linkshänder und 3 % Beidhänder, bei den Frauen 93 % Rechtshänder, 5 % Linkshänder und 2 % Beidhänder. Nach Korrektur für Alter und Geschlecht zeigten sich keine relevanten Unterschiede in der mittleren maximalen GS zwischen Links- und Rechtshändern (38,6 kg vs. 38,8 kg). Beidhänder, also Personen ohne eindeutig dominante Hand, zeigten eine leicht erhöhte mittlere maximale GS (39,2 kg). Bei der Mehrzahl der Teilnehmenden wurde der höchste Wert der GS-Messung an der dominanten Hand registriert (76 % der männlichen und 80 % der weiblichen Rechtshänder sowie 64 % der männlichen und 65 % der weiblichen Linkshänder). Die maximale GS der dominanten Hand lag bei männlichen Rechtshändern im Mittel 6 % (Frauen: 8 %) und bei männlichen Linkshändern 3 % (Frauen: 4 %) höher als die der nichtdominanten Hand.

Bei drei vorhandenen Messungen je Hand zeigte sich ein Anstieg der GS von der ersten zur dritten Messung. Von der ersten zur dritten Messung stieg der Mittelwert auf der rechten Seite von 34,8 kg auf 36,1 kg, auf der linken Seite von 33,2 kg auf 33,5 kg.

### Kardiorespiratorische Fitness

Insgesamt 46,2 % der Teilnehmenden mit auswertbarer Fahrradergometriemessung erreichten bzw. überschritten die angestrebte submaximale HF-Schwelle von 85 % der maximalen HF. Die Schwelle von 75 % der maximalen HF wurde von 82 % der Teilnehmenden erreicht bzw. überschritten.

**Abb. 2:**
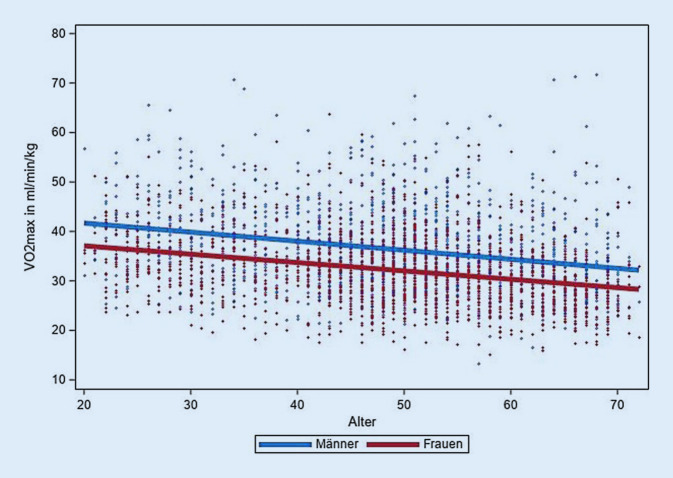
Zusammenhang zwischen Alter und maximaler Sauerstoffaufnahme (VO_2max_ in ml·min^−1^ · kg^−1^). Scatterplot mit geschlechtsspezifischer linearer Regressionsgerade. Daten der NAKO Gesundheitsstudie zur Halbzeit der Basiserhebung

Die HF-bezogenen Leistungsparameter der CRF, PWC 75 % und VO_2max_, zeigten die erwartete Alters- und Geschlechterverteilung (Tab. 3; Abb. 2 und 3). Bei Männern lag die altersadjustierte VO_2max_ durchschnittlich bei 36,4 ml·min^−1^ · kg^−1^ (PWC 75 % =1,65 W/kg), Frauen hatten eine mittlere VO_2max_ von 32,3 ml·min^−1^ · kg^−1^ (PWC 75 % = 1,32 W/kg). Bei der Altersgruppe ≥60 Jahre wurde eine um 19 % geringere VO_2max_ bzw. eine um 27 % geringere PWC 75 % als in der Altersgruppe 20–29 Jahre beobachtet.

**Tab. 3:** Kardiorespiratorische Fitness nach Alter und Geschlecht. Daten der NAKO Gesundheitsstudie zur Halbzeit der Basiserhebung

	Männer			Frauen		
Altersgruppen	*N*	PWC 75 % in W/kg (SD)	VO_2max_ in ml · min^−1^ · kg^−1^ (SD)	*N*	PWC 75 % in W/kg (SD)	VO_2max_ in ml · min^−1^ · kg^−1^ (SD)
20–29	133	1,95 (0,45)	40,81 (9,29)	173	1,54 (0,36)	35,99 (6,39)
30–39	186	1,83 (0,44)	38,63 (8,75)	196	1,46 (0,38)	34,27 (7,47)
40–49	397	1,69 (0,41)	37,03 (8,25)	422	1,36 (0,38)	32,79 (7,22)
50–59	417	1,61 (0,42)	35,89 (8,29)	482	1,30 (0,38)	32,05 (7,20)
≥60	330	1,42 (0,41)	33,08 (7,93)	358	1,12 (0,37)	29,09 (6,96)
*Gesamt*	1463	1,65 (0,45)	36,36 (8,64)	1631	1,32 (0,40)	32,28 (7,39)

*PWC 75* *%* Leistung in W/kg Körpergewicht bei Erreichen von 75 % der maximalen Herzfrequenz, *VO*_*2max*_ maximale Sauerstoffaufnahme, *SD* Standardabweichung, *N* Anzahl Beobachtungen

**Abb. 3:**
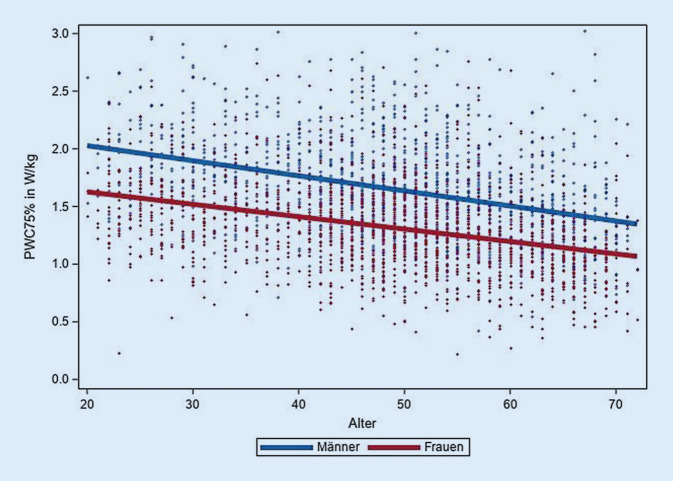
Zusammenhang zwischen Alter und der Leistung bei Erreichen von 75 % der maximalen Herzfrequenz (PWC 75 % in Watt pro kg Körpergewicht). Scatterplot mit geschlechtsspezifischer linearer Regressionsgerade. Daten der NAKO Gesundheitsstudie zur Halbzeit der Basiserhebung

### Zusammenhang von maximaler Handgreifkraft und kardiorespiratorischer Fitness

**Abb. 4:**
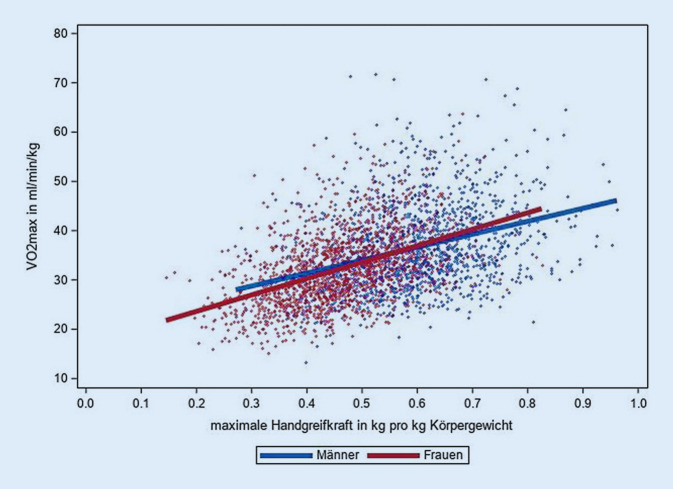
Zusammenhang der maximalen Handgreifkraft (in kg pro kg Körpergewicht) mit der maximalen Sauerstoffaufnahme (VO_2max_). Scatterplot mit geschlechtsspezifischer linearer Regressionsgerade. Daten der NAKO Gesundheitsstudie zur Halbzeit der Basiserhebung

Die Analyse des Zusammenhangs der maximalen GS mit der CRF zeigte eine positiv lineare Assoziation beider Parameter der körperlichen Fitness (Abb. 4). Der Anstieg der GS um 1 kg war bei Männern nach Korrektur für das Körpergewicht mit einem durchschnittlichen Anstieg der VO_2max_ von β = 0,21 ml·min^−1^ · kg^−1^ (Standardfehler SE = 0,03) assoziiert (Frauen β = 0,35 ml · min^−1^ · kg^−1^, SE = 0,03). Der Korrelationskoeffizient der für das Körpergewicht korrigierten maximalen GS mit der VO_2max_ betrug bei Männern r = 0,35, bei Frauen r = 0,46.

## Diskussion

Die Muskelkraft und die CRF sind die wesentlichen Merkmale der körperlichen Fitness und damit von großer Bedeutung als Risikofaktoren für Morbidität und Mortalität [2, 8]. Für eine genaue Abschätzung der Effekte der körperlichen Fitness auf gesundheitsrelevante Endpunkte ist eine möglichst präzise Erfassung der körperlichen Fitness notwendig. Insbesondere in großen, bevölkerungsbezogenen Studien zur Erforschung der Ätiologie von nichtübertragbaren Erkrankungen, in denen multikausale Modelle untersucht werden, muss aus Gründen der Machbarkeit aber häufig auf einfache, nichtinvasive, die Teilnehmenden wenig belastende Untersuchungsmethoden zurückgegriffen werden. In der NAKO wurde daher viel Wert auf die Auswahl geeigneter Erhebungsinstrumente u. a. zur Erfassung der körperlichen Fitness gelegt [30]. In der vorliegenden Arbeit wurden die in der NAKO angewandten Methoden zur Erfassung der körperlichen Fitness ausführlich beschrieben und erstmals deskriptive Ergebnisse aus einer Teilstichprobe berichtet.

Die Erfassung der Muskelkraft erfolgte in der NAKO, wie in vergleichbaren Kohortenstudien, über die Messung der maximalen GS [31, 32]. Die Messung der GS mittels Handdynamometer ist ein einfach durchzuführender, gut standardisierter Test [18]. Die ersten deskriptiven Ergebnisse aus der NAKO bestätigen den in anderen Studien gezeigten Einfluss des Alters und Geschlechts auf die maximale GS, mit einem Rückgang der GS ab der 6. Altersdekade [33–35]. Die alters- und geschlechtsspezifische maximale GS liegt in der NAKO in einem Bereich, der auch in Studien anderer Industrienationen festgestellt wurde [35]. Der in unseren Daten beobachtete Zusammenhang der Handdominanz mit der maximalen GS zeigte sich auch in anderen Studien [34]. Es existieren jedoch auch Untersuchungen mit zumeist kleineren Fallzahlen, die keinen Einfluss der Handdominanz zeigen konnten [36, 37].

Im Gegensatz zur relativ einfachen Messung der maximalen GS, ist die Messung der CRF komplexer. Als Goldstandard gilt die Messung der VO_2max_ im Rahmen einer spiroergometrischen Ausbelastung [16]. Dieses Verfahren wird jedoch in großen bevölkerungsbezogenen Studien aufgrund der hohen zeitlichen und technischen Anforderungen, der Schwierigkeit, ungeübte Teilnehmende wirklich auszubelasten, sowie aufgrund des im Vergleich zu submaximalen Tests höheren Risikos für kardiovaskuläre Ereignisse kaum eingesetzt [38]. Es existieren daher verschiedene Verfahren der Schätzung der VO_2max_ über die Ergebnisse aus submaximalen Ergometertests [16]. Das Testprotokoll, welches in der NAKO angewendet wird, wurde im Rahmen von Prätests entwickelt und erprobt [30] und zeigte in einer bisher unveröffentlichten Studie eine gute Validität der Schätzung der VO_2max_ im Vergleich zur Spiroergometrie (ICC = 0,79). Die ersten hier präsentierten Ergebnisse zur CRF aus einer Teilstichprobe der NAKO zeigen im Vergleich mit anderen populationsbasierten Studien plausible Ergebnisse hinsichtlich des Alters- und Geschlechtseffekts auf die CRF. Im Vergleich zu einer umfangreichen US-amerikanischen Studie zeigt sich in unserer Studie eine niedrigere VO_2max_ in den jüngeren Altersklassen (Männer 47,6 ml·min^−1^ · kg^−1^ vs. 40,8 ml·min^−1^ · kg^−1^ und Frauen 37,6 ml·min^−1^ · kg^−1^ vs. 36,0 ml·min^−1^ ·kg^−1^), jedoch eine höhere VO_2max_ in den höheren Altersklassen (29,4 ml·min^−1^ ·kg^−1^ vs. 33,1 ml·min^−1^ · kg^−1^ und 20,7 ml·min^−1^ · kg^−1^ vs. 29,1 ml·min^−1^ · kg^−1^; [39]). Die Autoren berichten einen Rückgang der VO_2max_ von 10 % pro Altersdekade bei Männern und Frauen, wohingegen der Rückgang in unserer Studie nur bei 6 % bzw. 5 % lag, was wiederum gut vergleichbar mit Daten der US-amerikanischen Cooper Center Longitudinal Study und auch der deutschen SHIP-Studie ist [40, 41]. Hinsichtlich der PWC 75 % zeigte sich im Vergleich zu Daten der „Studie zur Gesundheit Erwachsener in Deutschland“ (DEGS1) ein ähnlicher Rückgang zwischen der 3. und 6. Altersdekade [42]. Die im Vergleich zu DEGS leicht höheren Werte der CRF in der NAKO sind möglicherweise auf einen Selektionseffekt in der NAKO und den damit verbundenen höheren Anteil an gesünderen Teilnehmenden zurückzuführen. Generell ist für die Interpretation von leistungsbasierten Daten der CRF festzuhalten, dass vor allem Unterschiede im Testprotokoll (Laufband- vs. Fahrradergometertest etc.) und in der untersuchten Studienpopulation den Vergleich von alters- und geschlechtsspezifischen Mittelwerten der CRF einschränken [38].

Die Untersuchung des Zusammenhangs von GS und CRF in der NAKO zeigten eine moderate Korrelation mit Korrelationskoeffizienten zwischen 0,35 und 0,46. Vergleichbare Untersuchungen in der Studie „UK-Biobank“ im Vereinigten Königreich ergaben etwas stärkere Zusammenhänge von GS und CRF (r = 0,55), zeigten in weiterführenden Analysen aber die unabhängige Bedeutung der beiden Komponenten der körperlichen Fitness als Mortalitätsprädiktoren [43]. Prinzipiell lässt sich der Zusammenhang von Muskelkraft und CRF darüber erklären, dass insbesondere die Kraft der unteren Extremitäten, für welche die GS ein guter Indikator ist, das Ergebnis von CRF-Tests beeinflusst [44].

### Stärken und Limitationen

Die Stärke der Erfassung der körperlichen Fitness in der NAKO liegt vor allem in der Fallzahl, dem Bevölkerungsbezug und in der hohen Standardisierung der Messungen begründet. Durch die umfassende Phänotypisierung und die Verfügbarkeit von verschiedenen Biomaterialien für eine spätere Genotypisierung der Teilnehmenden hat der NAKO-Datensatz ein großes Potenzial für zukünftige, tiefergehende Analysen.

Einschränkend ist zu erwähnen, dass die hier gezeigten Ergebnisse aus einer Teilstichprobe der NAKO entstammen und Selektionseffekte die Ergebnisse der körperlichen Fitness beeinflusst haben können. Die gezeigten alters- und geschlechtsspezifischen deskriptiven Ergebnisse können daher auch nicht als Referenzdaten betrachtet werden. Erst nach Abschluss der Basisuntersuchung und unter Berücksichtigung weiterer Charakteristika von Teilnehmenden und Teilnahmeverweigerern können abschließende Aussagen zur körperlichen Fitness in der NAKO getroffen werden.

Die Erfassung der Muskelkraft erfolgte in der NAKO nur über den Proxy der maximalen GS. Verschiedene Untersuchungen zeigen jedoch, dass die GS ein guter Indikator für die generelle Muskelkraft ist [45, 46].

Der submaximale Ergometertest wurde nur bei einer Teilstichprobe der NAKO an 6 von 18 Studienzentren durchgeführt, was zu einer im Verhältnis zur Gesamtstichprobe kleinen Fallzahl geführt hat. Für Teilnehmende ohne Ergometertest kann die CRF weniger präzise anhand von nichtergometriebasierten Daten, wie Alter, Geschlecht, Gewicht, Ruheherzfrequenz und körperliche Aktivität, geschätzt werden [23, 47, 48]. Die NAKO bietet dabei durch die breite Datenerhebung die Option, entsprechende Modelle weiterzuentwickeln und anzuwenden.

Die Messung der CRF erfolgt in der NAKO aus Gründen der Machbarkeit nicht über den Goldstandard der Spiroergometrie, sondern über einen auf maximal vier Belastungsstufen begrenzten submaximalen Ergometertest, wobei in dieser Stichprobe nicht alle Teilnehmenden auch die Ziel-HF von 85 % der maximalen HF erreichten. Prinzipiell zeigen submaximale Belastungstests jedoch eine moderate bis hohe Genauigkeit in der Schätzung der CRF [16].

Die hier gezeigte Schätzung der CRF beruht auf der Messung der HF während der Belastung und der approximierten maximalen HF. Daher wurden Teilnehmende mit bekannter aktueller Einnahme von Betablockern aus der Analyse der CRF-Daten ausgeschlossen. Es ist jedoch nicht auszuschließen, dass hinsichtlich der Betablockereinnahme insbesondere in den höheren Altersgruppen falsch-negative Fälle zu einer Überschätzung der mittleren CRF geführt haben. Durch Hinzunahme weiterer, zukünftig zur Verfügung stehender Informationen (detaillierte Medikamentendokumentation) wird eine genauere Analyse von CRF-beeinflussenden Medikationen möglich sein. Bei Personen mit HF-beeinflussender Medikation erlaubt die Erfassung der RPE-Werte am Ende jeder Belastungsstufe für zukünftige Analysen die Schätzung der CRF über entsprechende Schätzgleichungen [16, 49].

## Fazit

Die körperliche Fitness wird in der NAKO umfassend bestimmt. Die vorliegenden ersten Ergebnisse zur körperlichen Fitness weisen auf eine hohe Plausibilität der erhobenen Daten im Vergleich zu Daten aus anderen populationsbasierten Studien hin und unterstreichen die Bedeutung der NAKO als Forschungsplattform für zukünftige Analysen zum besseren Verständnis der Entstehung von häufigen Erkrankungen. Mit der aktuell stattfindenden ersten Folgeuntersuchung der NAKO kann zukünftig nicht nur die einmalig gemessene körperliche Fitness berücksichtigt werden, sondern auch deren Veränderung im Zeitverlauf.
