# Erratum zu: Messung der körperlichen Fitness in der NAKO Gesundheitsstudie – Methoden, Qualitätssicherung und erste deskriptive Ergebnisse

**DOI:** 10.1007/s00103-025-04083-9

**Published:** 2025-07-11

**Authors:** Alexander Kluttig, Johannes Zschocke, Johannes Haerting, Axel Schmermund, Sylvia Gastell, Karen Steindorf, Florian Herbolsheimer, Andrea Hillreiner, Carmen Jochem, Sebastian Baumeister, Ole Sprengeler, Tobias Pischon, Lina Jaeschke, Karin B. Michels, Lilian Krist, Halina Greiser, Gerhard Schmidt, Wolfgang Lieb, Sabina Waniek, Heiko Becher, Annika Jagodzinski, Sabine Schipf, Henry Völzke, Wolfgang Ahrens, Kathrin Günther, Stefanie Castell, Yvonne Kemmling, Nicole Legath, Klaus Berger, Thomas Keil, Julia Fricke, Matthias B. Schulze, Markus Loeffler, Kerstin Wirkner, Oliver Kuß, Tamara Schikowski, Sonja Kalinowski, Andreas Stang, Rudolf Kaaks, Antje Damms Machado, Michael Hoffmeister, Barbara Weber, Claus-Werner Franzke, Sigrid Thierry, Anette Peters, Nadja Kartschmit, Rafael Mikolajczyk, Beate Fischer, Michael Leitzmann, Mirko Brandes

**Affiliations:** 1 Institut für Medizinische Epidemiologie, Biometrie und Informatik, Martin-Luther-Universität Halle-Wittenberg, Magdeburger Str. 8, 06112 Halle (Saale), Deutschland; 2 Institut für Physik, Martin-Luther-Universität Halle-Wittenberg, Halle (Saale), Deutschland; 3 Bethanien Hospital, Frankfurt, Deutschland; 4 NAKO Studienzentrum, Deutsches Institut für Ernährungsforschung, Potsdam-Rehbrücke, Deutschland; 5 Abteilung Bewegung, Präventionsforschung und Krebs, Deutsches Krebsforschungszentrum (DKFZ), Heidelberg, Deutschland; 6 Institut für Epidemiologie und Präventivmedizin, Universität Regensburg, Regensburg, Deutschland; 7 Lehrstuhl für Epidemiologie der LMU München, UNIKA-T, Augsburg, Deutschland; 8 BIPS, Leibniz Institut für Präventionsforschung und Epidemiologie, Bremen, Deutschland; 9 Forschergruppe Molekulare Epidemiologie, Max-Delbrück-Centrum für Molekulare Medizin in der Helmholtz-Gemeinschaft (MDC), Berlin, Deutschland; 10 Institut für Prävention und Tumorepidemiologie, Universitätsklinikum Freiburg, Medizinische Fakultät, Albert-Ludwigs-Universität Freiburg, Freiburg, Deutschland; 11 Institut für Sozialmedizin, Epidemiologie und Gesundheitsökonomie, Charité – Universitätsmedizin Berlin, Berlin, Deutschland; 12 Hochschule Kaiserslautern, Zweibrücken, Deutschland; 13 Institut für Epidemiologie, Christian-Albrechts-Universität Kiel, Kiel, Deutschland; 14 Institut für Medizinische Biometrie und Epidemiologie, Universitätsklinikum Hamburg-Eppendorf, Hamburg, Deutschland; 15 Epidemiologisches Studienzentrum, Universitätsklinikum Hamburg-Eppendorf, Hamburg, Deutschland; 16 Institut für Community Medicine, Universitätsmedizin Greifswald, Greifswald, Deutschland; 17 Helmholtz-Zentrum für Infektionsforschung (HZI), Braunschweig, Deutschland; 18 Institut für Epidemiologie und Sozialmedizin, Universität Münster, Münster, Deutschland; 19 Institut für Medizinische Informatik, Statistik und Epidemiologie (IMISE), Universität Leipzig, Leipzig, Deutschland; 20 Institut für Biometrie und Epidemiologie, Deutsches Diabetes-Zentrum (DDZ), Leibniz-Zentrum für Diabetes-Forschung an der Heinrich-Heine-Universität Düsseldorf, Düsseldorf, Deutschland; 21 IUF – Leibniz-Institut für umweltmedizinische Forschung, Düsseldorf, Deutschland; 22 Institut für Medizinische Informatik, Biometrie und Epidemiologie (IMIBE), Universitätsklinikum Essen, Essen, Deutschland; 23 Institut für Epidemiologie, Helmholtz Zentrum München, Neuherberg, Deutschland; 24 LIFE – Leipziger Forschungszentrum für Zivilisationserkrankungen, Universität Leipzig, Leipzig, Deutschland; 25 Institut für Statistik, Fachbereich Mathematik und Informatik, Universität Bremen, Bremen, Deutschland; 26 Charité – Universitätsmedizin Berlin, Berlin, Deutschland; 27 MDC/BIH Biobank, Max-Delbrück-Centrum für Molekulare Medizin in der Helmholtz-Gemeinschaft (MDC) und Berlin Institute of Health (BIH), Berlin, Deutschland; 28 Partnerstandort Berlin, Deutsches Zentrum für Herz-Kreislauf-Forschung (DZHK), Berlin, Deutschland; 29 Abteilung Molekulare Epidemiologie, Deutsches Institut für Ernährungsforschung, (DIfE), Nuthetal, Deutschland; 30 Institut für Klinische Epidemiologie und Biometrie, Universität Würzburg, Würzburg, Deutschland; 31 Landesinstitut für Gesundheit, Bayerisches Landesamt für Gesundheit und Lebensmittelsicherheit, Bad Kissingen, Deutschland; 32 Krebsregister Saarland, Saarbrücken, Deutschland; 33 Deutsches Krebsforschungszentrum (DKFZ), Heidelberg, Deutschland; 34 Selbstständige Forschungsgruppe Klinische Epidemiologie, Helmholtz Zentrum München, Deutsches Forschungszentrum für Gesundheit und Umwelt, München, Deutschland; 35 Abteilung Klinische Epidemiologie und Alternsforschung, Deutsches Krebsforschungszentrum (DKFZ), Heidelberg, Deutschland; 36 Partnerstandort Greifswald, Deutsches Zentrum für Herz-Kreislauf-Forschung (DZHK), Greifswald, Deutschland; 37 Partnerstandort Hamburg, Deutsches Zentrum für Herz-Kreislauf-Forschung (DZHK), Hamburg, Deutschland

**Erratum zu:**

**Bundesgesundheitsbl 2020**

10.1007/s00103-020-03100-3

In diesem Artikel hätte der Satz „Mit der geschätzten maximalen Leistung wurde über die Formel des American College of Sports Medicine [29] die VO_2max_ berechnet: VO_2max_ = 33,5 ml·min^−1^·kg^−1^ + 12,24 (maximale Leistung in W) (Körpergewicht^−1^ in kg).“ richtigerweise „Mit der geschätzten maximalen Leistung wurde über eine modifizierte und im Rahmen eines Prätests validierten Formel des American College of Sports Medicine [29] die VO_2max_ berechnet: VO_2max_ = 3,5 ml·min^−1^·kg^−1^ + 12,24 (maximale Leistung in W) (Körpergewicht^−1^ in kg).“ lauten sollen.

Der Originalbeitrag wurde korrigiert.

